# Diet Quality and Sociodemographic, Lifestyle, and Health-Related Determinants among People with Depression in Spain: New Evidence from a Cross-Sectional Population-Based Study (2011–2017)

**DOI:** 10.3390/nu13010106

**Published:** 2020-12-30

**Authors:** Jesús Cebrino, Silvia Portero de la Cruz

**Affiliations:** 1 Department of Preventive Medicine and Public Health, Faculty of Medicine, University of Seville, Avda. Doctor Fedriani S/N, 41009 Seville, Spain; jcebrino@us.es; 2 Department of Nursing, Pharmacology and Physiotherapy, Faculty of Medicine and Nursing, University of Córdoba, Avda. Menéndez Pidal S/N, 14071 Córdoba, Spain

**Keywords:** depressive disorder, diet, mental disorders, nutrition surveys, population, trends

## Abstract

The role of diet quality in depression is an emerging research area and it appears that diet quality could be an important modifying factor. The aims of this study were to report the prevalence of diet quality among individuals with and without a self-reported diagnosis of depression aged from 16 to 64 years old in Spain, to analyze the time trends of the frequency of food consumption and diet quality from 2011 to 2017 in individuals with a self-reported diagnosis of depression, and to explore the associations between poor/improvable diet quality and sociodemographic, lifestyle, and health-related factors. A nationwide cross-sectional study was conducted in 42,280 participants with and without a self-reported diagnosis of depression who had participated in the 2011/2012 and 2017 Spanish National Health Surveys and the 2014 European Health Survey in Spain. A logistic regression analysis was performed to identify the variables associated with diet quality. The overall prevalence of diet quality among depressive and non-depressive individuals revealed 65.71% and 70.27% were in need of improvement, respectively. Moreover, having a poor or improvable diet quality is associated with male gender, people aged 16–24 years old and 25–44 years old, separated or divorced, and also in smokers.

## 1. Introduction

Depression ranks globally among the top 10 disability causes [1,2], affecting approximately 5% to 6% of people each year worldwide and 11% to 15% of people in their lifetime [3]. Moreover, depression is often recurrent or chronic and has a negative impact on people’s functioning and somatic health [4]. Additionally, it has been associated with a poor quality of life, physical decline, higher risk of premature death, and a greater economic burden [5]. Thus, depression is an important public health concern, for which there is an urgent need to identify modifiable factors to reduce its prevalence [6].

The role of diet quality in mental health illnesses and, in particular, depression is an emerging research area, and it appears that diet quality could be a major modifying factor [7,8,9,10]. Although some authors have found no associations [8], others show a link between a healthy dietary pattern and a reduced likelihood of depressive symptoms [7,11]. However, direct evidence is not readily available [12,13,14].

Some studies have found that depressive symptoms were associated with higher intakes of sugar, sodium, and saturated fat [15,16] or frequent consumption of meat and eggs [9,17] and lower intakes of low-calorie foods [18] and antioxidants, fruit and vegetables [19], fish [9], or legumes [20]. However, further studies are needed to assess the influence of types of diet on depression [21]. For example, the impact of soft drinks on mental health has drawn considerable interest from researchers in recent years due to the fact that numerous studies have suggested a consistent association between soft drink consumption and depressive symptoms [22,23,24].

It seems clear that the factors associating depression with diet quality include sociodemographic and economic conditions. For example, depression is more prevalent in people with low socioeconomic status [25,26], probably due to the fact that higher diet quality or “healthy foods” often have limited uptake because they are more costly [27,28]. In addition, any observed association between depression and diet quality might be accounted for by lifestyle habits, given that people with depression engage in less leisure-time physical activity than those without depression [29], due to a lack of energy and greater fatigue, which are common symptoms of depression [30]. Moreover, the lack of interest in various activities, for example, the motivation to cook or enjoy meals, may be explained by depressive symptoms [31].

Nowadays, drugs and psychological interventions are used to reduce symptoms of depression. Nevertheless, psychological interventions only reduce the incidence rate of depression by 20–25% [32] and medications show minimal benefits in the sub-threshold of depression [33]. Considering the rise in the number of people with depression worldwide [34], preventive strategies are needed in order to reduce its prevalence. For this reason, it is important to reflect on numerous factors affecting the development of depression [35], with particular attention given to modifiable behavior such as a diet that can potentially prevent this disorder [36]. In recent years, there has been a shift of focus from studying single nutrients toward dietary patterns [37]. This particular study uses diet quality scores to evaluate dietary patterns, based on the dietary guidelines of the Spanish Society of Community Nutrition (SSCN) [38]. Moreover, this study is the first to show the relationship between numerous different sociodemographic, lifestyle, and health-related characteristics and diet quality independently and simultaneously in a large, representative sample of the population with a self-reported diagnosis of depression in Spain, aged between 16–64 years old, conducted from 2011 to 2017. Therefore, the main objectives of the present study were to report the prevalence of diet quality among individuals with and without a self-reported diagnosis of depression aged from 16 to 64 years old in Spain, to analyze the time trends of the frequency of food consumption and diet quality from 2011 to 2017 in individuals with a self-reported diagnosis of depression, and to explore the associations between poor/improvable diet quality and sociodemographic, lifestyle, and health-related factors.

## 2. Materials and Methods

### 2.1. Study Design

A quantitative, observational, nationwide, cross-sectional study.

### 2.2. Data Source and Study Population

The data were obtained from the personalized interviews in the Spanish National Health Survey (SNHS) 2011/2012 (from July 2011 to July 2012) [39], the European Health Survey in Spain (EHSS) 2014 (from January 2014 to January 2015) [40], and the SNHS 2017 (from October 2016 to October 2017) [41]. The SNHS and EHSS had a cross-sectional and population-based design and were conducted at the national level, focusing on the non-institutionalized population (representativeness is ensured by assigning a weighting coefficient to each participant), through an interview. These surveys were carried out by the National Institute of Statistics and the Ministry of Health, Consumer Affairs, and Social Welfare in Spain. These personal interviews were multistage probabilistic, stratified sampling by census areas (first stage), sections (second stage), and individuals (third stage). The selected households were initially contacted through a letter from the Ministry of Health, Consumer Affairs, and Social Welfare in Spain requesting their collaboration, in which they were informed that they had been selected for the survey and that this survey was confidential and they were notified of the upcoming visit of a duly authorized interviewer. A detailed description of SNHS and EHSS methodologies can be found elsewhere [39,40,41].

**Figure 1:**
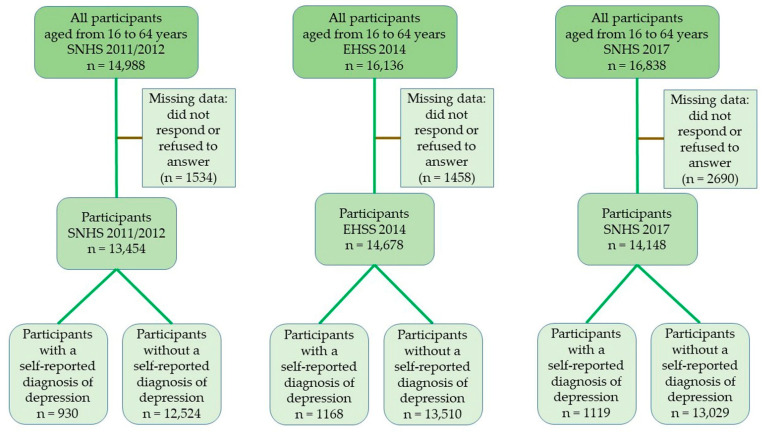
Flowchart of the study population. SNHS: Spanish National Health Survey 2011/2012 and 2017; EHSS: European Health Survey in Spain 2014.

For the data analyzed, the inclusion criteria were: people aged from 16 to 64 years old, who were resident in Spain during the years of the surveys. Figure 1 shows the flowchart of the study population. From the initial 47,962 participants (SNHS 2011/2012: n = 14,988; EHSS 2014: n = 16,136; SNHS 2017: n = 16,838), we excluded 5682 individuals who did not respond or refused to answer the interview questions (SNHS 2011/2012: n = 1534; EHSS 2014: n = 1458; SNHS 2017: n = 2690). For the cross-sectional analysis, we included 3217 participants with a self-reported diagnosis of depression (SNHS 2011/2012: n = 930; EHSS 2014: n = 1168; SNHS 2017: n = 1119) and 39,063 without a self-reported diagnosis of depression (SNHS 2011/2012: n = 12,524; EHSS 2014: n = 13,510; SNHS 2017: n = 13,029).

For the purpose of the current study, we assessed the presence of depression through the health status module of an adult questionnaire from SNHS 2011/2012 [39] and 2017 [41] and EHSS 2014 [40]. The adult questionnaire collects individual information on a person aged 15 and over (for SNHS 2011/2012 and SNHS 2017) and 16 and over (for EHSS 2014). This information covers all the survey’s health variables and is structured into three modules: (i) health status module, (ii) healthcare module, and (iii) health determinants module. The health status module collects information on perceived health status, chronic disease and limitation, diseases and health problems, accidents, restriction of activity, physical, sensory, and cognitive limitations, limitations on daily activities, mental health, stress, and job satisfaction. We identified individuals suffering from depression as those that answered “yes” to the question “Have you ever been diagnosed depression by a physician?”.

### 2.3. Variables

#### 2.3.1. Diet Quality

The dependent variable was diet quality. This variable was measured using the Spanish Health Eating Index (SHEI) [42]. This instrument was developed to measure how well diets meet the food-based dietary guidelines of the Spanish Society of Community Nutrition (SSCN) [38] and contain 10 items that represent food groups from the dietary guidelines. Each variable represents: (i) bread or grains, (ii) leafy greens, salads, and vegetables, (iii) fresh fruit (excluding juices), (iv) dairy products (milk, cheese, yoghurt), (v) meat (chicken, beef, pork, lamb, etc.), (vi) legumes, (vii) cold meats and cuts, (viii) sweets (biscuits, pastries, jams, cereals with sugar, sweets, etc.), (ix) soft drinks with sugar, and (x) variety of the diet, built on the recommendations of SSCN. These items were identically worded in the questionnaires and identical in the SNHS 2011/2012 and 2017, and EHSS 2014. Each of the items is divided into 5 categories, which refer to the frequency of food consumption: never or hardly ever, less than once a week, once or twice a week, three or more times a week, but not daily, and daily. The food groups were categorized as follows: bread or grains, leafy greens, salads and vegetables, fresh fruit (excluding juices), dairy products (milk, cheese, yogurt), which represent the food groups for daily consumption; meat (chicken, beef, pork, lamb, etc.) and legumes correspond to the weekly consumption food groups; cold meats and cuts, sweets (biscuits, pastries, jams, cereals with sugar, sweets, etc.) and soft drinks with sugar correspond to the occasional food groups; and the last represents the variety of the diet, a fundamental objective in a healthy diet. Each food group received a score, which ranged from 0 to 10 according to the criteria established in the Supplementary Table S1, where 10 points in a food group means that it complies with the recommendations proposed by the Spanish Society of Community Nutrition [38]. Total (overall) SHEI scores range from 0 to 100 and are the sum of the frequency of consumption of 10 food groups. The SHEI result contains three categories: poor diet, diet in need of improvement, and good diet, using the cut-off points previously established in the questionnaire validation [42]: poor diet quality (SHEI score <51), diet in need of improvement (SHEI score between 51 and 80), and good diet quality (SHEI score >80).

#### 2.3.2. Sociodemographic Variables

The independent variables were: year of the surveys (2011/2012, 2014, 2017), gender (female, male), age group (16–24 years, 25–44 years, 45–64 years), marital status (single, married, widowed, separated/divorced), level of education (without studies, primary, secondary or professional training, university), nationality (Spanish, foreign), and size of the town of residence (<10,000 inhabitants, ≥10,000 inhabitants).

Social class, as an independent variable, was assigned according to the categories proposed by the Spanish Society of Epidemiology [43]. This variable was classified into: Class I (directors and managers of companies with 10 or more employees and professionals normally qualified with university degrees), Class II (directors and managers of companies with less than 10 salaried employees and professionals normally qualified with university degrees and other technical support professionals. Athletes and artists), Class III (intermediate professions and self-employed workers), Class IV (supervisors and workers in skilled technical work), Class V (skilled workers in the primary sector and other semi-skilled workers), Class VI (unskilled workers). For the purposes of this study, these six original classes were rearranged into three groups (Classes I and II, Classes III and IV, Classes V and VI).

#### 2.3.3. Health-Related Variables

Body mass index (BMI), which was calculated from the self-reported values of body weight and height, was classified according to the World Health Organization [44]. Thus, the following categories were used: underweight (BMI <18.50 kg/m^2^), normal-weight (BMI ranged between 18.50 and 24.99 kg/m^2^), overweight (BMI ranged between 25.00 and 29.99 kg/m^2^), and obesity (BMI ≥30 kg/m^2^).

Other health-related variables in the study were: current smoking habit (yes, no), consumption of alcoholic beverages in the past 12 months prior to the survey (yes, no), and self-perceived health status (very good, good, fair, poor, very poor).

#### 2.3.4. Lifestyle Behavior

Lifestyle behavior included: physical activity in main activity (physically active in the main activity, not physically active in main activity), physical activity during leisure time (yes, no), and the number of days in the last 7 days when the respondent walked for at least 10 min at a time (maximum 7 days).

### 2.4. Ethical Aspects

The data obtained from these surveys are available on the Ministry of Health, Consumer Affairs, and Social Welfare of Spain and the National Institute of Statistics websites [39,40,41] in the form of anonymized microdata: no special authorization is, therefore, required for their use. According to the SNHS and EHSS methodology, the microdata files are stored anonymously and are available to the public. In accordance with Spanish law, when secondary data are used, there is no need for approval from an Ethics Committee. The research data is available here as a Supplementary File.

### 2.5. Statistical Analysis

A descriptive analysis was performed by calculating the counts and percentages for the qualitative variables and the continuous variables by calculating the arithmetic mean and standard deviation (SD). Sociodemographic, lifestyle, health-related characteristics, and the diet quality of people with and without a self-reported diagnosis of depression were compared using the Chi-square test for contingency tables or Fisher’s exact test if the number of expected frequencies was greater than 5. For the bivariate analysis, Student’s t-test for means in normal distribution variables was used. Linear regression models were used to identify statistically significant trends in the frequency of food consumption in the period of 2011–2017. The regression coefficient and the coefficient of determination (R^2^) were calculated to assess the direction, average magnitude of the change, and performance of the models. In addition, logistic regression was performed to identify the variables associated with the diet quality of people with a self-reported diagnosis of depression. It should be noted that the variable for diet quality of people with a self-reported diagnosis of depression was classified as “good diet” (a score over 80 on the SHEI) and “poor/improvable diet” (a score less than or equal to 80) for the bivariate and multivariate analysis. All the variables with a significant association in the bivariate analysis were included in the multivariate analysis. Crude and adjusted Odds Ratios (OR) were calculated with 95% confidence intervals. The Wald statistic was used to exclude one by one from the model any variables with a *p* ≥ 0.15 (backward methodical selection procedure). The goodness of fit was verified with the Hosmer–Lemeshow test. All the hypothesis contrasts were bilateral and in all the statistical tests with a 95% confidence level (*p* < 0.05) were considered significant values. The statistical power for all the analyses conducted was 80%. The variables that were part of the final multivariate-adjusted model were gender, age group, marital status, current smoking habit, consumption of alcoholic beverages in the past 12 months prior to the survey, and number of days in the last 7 days when the respondent had walked for at least 10 min at a time. The statistical analysis was carried out using IBM SPSS Statistics version 25 (IBM Corp, Armonk, NY, USA), licensed to the University of Seville (Spain).

## 3. Results

### 3.1. Sociodemographic, Lifestyle Habits and Health-Related Variables

The total number of individuals with a self-reported diagnosis of depression included in the study was 3217. Participants with a self-reported diagnosis of depression were more often females (68.67% vs. 48.97%, *p* < 0.001), 45–64 years old (72.09% vs. 45.57%, *p* < 0.001), Spanish (95.37% vs. 91.62%, *p* < 0.001), current smokers (37.24% vs. 31.09%, *p* < 0.001), and not physically active (86.23% vs. 80.42%, *p* < 0.001) compared to people without a self-reported diagnosis of depression (Table 1).

### 3.2. Diet Quality

As can be seen in Table 2, the prevalence of daily consumption of leafy greens, salads and vegetables, and fresh fruit was higher among participants with a self-reported diagnosis of depression (44.33% vs. 43.67% *p* < 0.001; 61.95% vs. 60.72% *p* < 0.001, respectively). Nonetheless, the daily consumption of bread was lower in that population (82.47% vs. 83.28% *p* < 0.001). Regarding diet quality, the prevalence of poor and good diet quality was higher in individuals with depression (3.05% vs. 2.71% *p* < 0.001; 31.24% vs. 27.02% *p* < 0.001, respectively). In addition, a diet in need of improvement was more prevalent in participants without a diagnosis of depression (70.27% vs. 65.71% *p* < 0.001).

According to the year of the survey (Table 3), there was a decrease in the number of people with a self-reported diagnosis of depression who never or hardly ever consumed legumes (β = −0.42, R^2^ = 1.00, *p* = 0.03). In the same way, the percentage of people with depression who consumed soft drinks with sugar on a daily basis decreased (β = −0.44, R^2^ = 1.00, *p* = 0.02).

### 3.3. Association between Sociodemographic, Lifestyle, and Health-Related Characteristics and Diet Quality

As regards the adjusted logistic regression model, Table 4 showed that the probability of having a poor or improvable diet quality was higher in males (OR = 1.47, 95% CI 1.23–1.76), people aged 16–24 years old (OR = 3.05, 95% CI 1.24–6.95) and 25–44 years old (OR = 2.32, 95% CI 1.89–2.86), separated or divorced (OR = 1.41, 95% CI 1.13–1.75), and people who currently smoked (OR = 1.70, 95% CI 1.43–2.02). In addition, the probability of having poor or improvable diet quality was lower in people who had not consumed alcoholic beverages in the past 12 months (OR = 0.80, 95% CI 0.69–0.94). Moreover, walking in the last 7 days for at least 10 min at a time was associated with diet quality (OR = 0.95, 95% CI 0.92–0.97).

## 4. Discussion

### 4.1. Main Findings

The present study, based on a large, representative population with a self-reported diagnosis of depression in Spain, between 16–64 years old, is the first to show the relationship between sociodemographic, lifestyle and health-related characteristics and diet quality from 2011 to 2017.

The results showed that diet quality among people with a self-reported diagnosis of depression living in Spain was largely improvable. This supports the view that people with depression have lower scores on healthy dietary pattern surveys [10,45,46]. For example, Spanish people belonging to the fast food consumption quintiles Q2 to Q5 showed an increased risk of depression compared to those participants belonging to the lowest level of fast food consumption [47]. These findings help improve our understanding of whether diet quality should be a novel intervention target for the primary prevention of depression [48]. Numerous meta-analyses and systematic reviews showed the connection between adherence to good diet quality and as much as a 33% lower risk of incident depressive outcomes [31,49,50,51,52]. As part of the SMILES trial, Jacka et al. [53] showed substantial improvements in symptoms of depression following seven consultations on healthy dieting. Nevertheless, it seems that selectively-induced expectancy and a loss of blinding may have contributed to the observed effect [54].

In the present study, it was found that the male gender was associated with an increased probability of having poor or improvable diet quality than women, an outcome which is consistent with other research [55,56,57]. One reason for this might be that women are more likely than men to make food choices for their health benefits or to maintain a lower body weight [58]. In addition, some studies reporting lower values associated with diet quality in men could be explained by a social perception that healthy eating is an inherently feminine habit [59,60,61,62]. Tailored dietary interventions targeted specifically towards men are needed to alter these social and gender norms that link masculinity with less healthy eating [63]. In particular, it is necessary for young men to learn healthy eating habits [64].

Young people are less likely to consume healthy food compared to other age groups [65,66]. This study confirmed that the younger the subject, the greater the risk of having a poor or improvable diet quality. For instance, Nour et al. [67] reported that young people aged from 18 to 24 years old reported less variety in the consumption of vegetables than people aged between 25 and 34. Moreover, older people are more likely to achieve the recommended daily consumption of fruit and vegetables than young people [68]. Interestingly, the literature shows that young people with lower diet quality scores were more likely to report depressive symptoms [69,70].

Confirming the findings from previous studies [71,72], our results showed that being divorced or separated was associated with a lower dietary diversity score. As shown in this study, the probability of having a poor or improvable diet quality was higher in this group. This may be due to limited financial resources and lack of family support, which may restrict their access to a variety of food choices [71]. Another possible explanation might be food insecurity, which is more prevalent in divorced or separated people [73].

Among the people who met the physical activity guidelines, people who had depression had a significantly lower probability of having a higher diet quality than people without [74]. This study found that having walked more days in the last 7 for at least 10 min at a time was associated with diet quality. The reasons for this could include personal and environmental factors, such as social support, accessibility, and the availability of healthy food choices, as well as the availability of physical exercise facilities and the opportunities for walking in the neighborhood [75,76]. It should be noted that exercise could be effective psychotherapy or alternative treatment for depression [77,78,79]. In fact, aerobic exercise at least 3 times per week, at a moderate to high-intensity, can significantly reduce depressive symptoms [80,81]. Therefore, healthy eating habits and increased physical activity are particularly promising targets [82], which have progressively featured in the clinical practice guidelines for managing depression [83].

According to population studies, smokers and non-smokers differ in the type of food they consume [84,85,86]. In fact, a meta-analysis that analyzed the links between smoking and diet has revealed that the dietary habits of smokers are characterized by higher intakes of energy, saturated fat, cholesterol, and alcohol and by lower intakes of vitamins, antioxidants, and fiber, in comparison with non-smokers [87]. Moreover, a number of studies have found that less fruit and vegetables were consumed by smokers than non-smokers [88,89]. The findings from this study also showed that the probability of having a poor or improvable diet quality was higher in people who currently smoked, as is also reflected in the extensive body of literature [88,90,91,92]. As regards alcohol consumption, the probability of having a poor or improvable diet quality was lower in people who had not consumed alcoholic beverages in the past 12 months. This difference in diet quality among consumers and non-consumers of alcoholic beverages was also found in other studies [93,94]. Alcohol is commonly consumed around mealtimes [95], and different habits of alcoholic beverage consumption were regularly associated with less varied diet quality [96] and its increased consumption associated with depression [97,98,99]. Therefore, this consumption behavior may act as a confounder, which may account for the links observed between diet quality and depression [100].

The scientific literature has found an association between soft drinks and depressive symptoms from adolescence to adulthood [23,101,102]. In addition, soft drink consumption has been linked to an increased risk of type 2 diabetes [103], cardiovascular disease [104], dental caries [105,106], and weight gain [107]. Reducing this type of consumption is, therefore, a high public health priority [108]. This study revealed that the percentage of people with a self-reported diagnosis of depression who consumed soft drinks with sugar daily had decreased. This decrease could be explained by the World Health Organization’s recommendation [109] to governments to reduce the consumption of products that are harmful to health through taxation and other policies in developed countries, including Spain [110]. This study also revealed that people with a self-reported diagnosis of depression who never or hardly ever consumed legumes had also decreased. For example, when legume consumption decreased among US adults, improved communication about their benefits was introduced [111].

We also identified some differences in dietary habits between depressed and non-depressed individuals. In that sense, the consumption of legumes once or twice a week was more frequent in individuals with no depression. Another study found a similar result [20]. This food group is rich in tryptophan, inositol, magnesium, and other important nutrients, such as fiber, folate, and omega-3 fatty acids. A previous study established a beneficial effect of the consumption of tryptophan, inositol, and magnesium on the mental well-being of individuals [112]. Additionally, this study showed that participants with depression had significantly higher daily consumption of sweets than their non-depressed counterparts. This finding is in line with the results of another study [113], which may be attributable to the sugar contents of this group of food. Sweets contain large amounts of sugars, which are associated with a high glycemic load [114]. Actually, research shows that high glycemic load diets are associated with a high level of pro-inflammatory cytokines and a worse lipid profile, which have already been proven to be related to high depressive symptoms [115]. In the present study, the prevalence of daily consumption of leafy greens, salads and vegetables, and fresh fruit was higher among participants with a self-reported diagnosis of depression. However, a case-control study carried out by Payne et al. [19] showed that depressed individuals consumed less fruit and vegetables than non-depressed. Regarding diet quality, observational studies have shown poorer diet quality in depressed versus non-depressed individuals, although null findings are common as well [116]. This result is similar to that found in the current study. The association of depression with poorer diets could be due to the appetite modification that frequently occurred after the disease development. Modification of appetite is a common symptom among those diagnosed with major depression, and it is one of the diagnostic criteria of depression in the DSM-V [45].

### 4.2. Strengths and Limitations

This study has certain limitations. Firstly, due to the cross-sectional design, it was not possible to assign causality between the sociodemographic, lifestyle, and health-related factors and diet quality. Secondly, a self-reported diagnosis of depression was used as a proxy for a confirmed diagnosis. Thirdly, it should be noted that no distinctions have been made between patients with different subtypes of depression. Moreover, people aged over 65 years were not included in this research, and therefore, the sample was not representative of all people living in Spain. Due to the fact that data from SNHS and EHSS are stored anonymously, it is impossible to know if a participant has taken part in more than one survey. Finally, it was not possible to separate remitted and current depressed subjects because neither SNHS nor EHSS took this aspect into account. On the other hand, one strength of our study is that since the data were derived from a national survey, they were obtained using a carefully planned methodology, including sampling, well-designed forms, preparation of the survey participants, supervision of the survey, and filtering of the data, all of which guarantee a representative sample of the population between 16–64 years old and lead to a greater understanding of this problem in today’s society. Moreover, the data information was collected by a trained interviewer from a personal interview, which avoids the other potential biases commonly found in telephone surveys.

### 4.3. Implications for Research and Practice

This large, representative sample of people with a self-reported diagnosis of depression in Spain between 16 and 64 years old enabled us to evaluate a vast number of associations with factors from different domains simultaneously. Thus, this study provides valuable insights that will be useful for conducting future research. Our results of the overall prevalence of diet quality revealed that 31.24% had a good diet quality and 65.71% were in need of improvement. It is vital for health authorities to take these findings into consideration when designing strategies to improve diet quality among individuals with depression. Although this research showed that the number of people who never or hardly ever consumed legumes and people who consumed soft drinks with sugar on a daily basis declined from 2011 to 2017, government agencies should persevere with their efforts to reduce the consumption of soft drinks with sugar due to its potential dangers for general health [117] and encourage people to consume more legumes for their health benefits [111]. Additionally, our findings of depressive people suggest that males, people aged 16–24 years old and 25–44 years, separated or divorced, and also smokers were more likely to have a poor or improvable diet quality. Therefore, Spanish mental health policies should be specially adapted to take these characteristics of the population into account in order to implement, for example, programs promoting a healthy diet [5]. Finally, further studies are needed to focus on how diet quality is mechanistically connected to depression, and on how to set up controls for the commonest confounders, such as exposure to stress; new experimental methods are also needed to study the effects of diet quality and their consequences for a population with depression [31].

## 5. Conclusions

The overall prevalence of diet quality among individuals with a self-reported diagnosis of depression in Spain showed that 65.71% were in need of improvement and 31.24% had a good diet quality. Among the individuals suffering from depression, there was a decrease from 2011 to 2017 in the number of people who never or hardly ever consumed legumes and people who consumed soft drinks with sugar on a daily basis. Having a poor or improvable diet quality is associated with male gender, people aged 16–24 years old and 25–44 years old, separated or divorced, and also in smokers. However, the likelihood of having a poor or improvable diet quality decreases in people who had not consumed alcoholic beverages in the past 12 months. Finally, walking in the last 7 days for at least 10 min at a time is associated with diet quality.

## Figures and Tables

**Table 1:** Sociodemographic, lifestyle, and health-related variables according to depressive status in people aged from 16 to 64 years (N = 42,280) (2011–2017).

Variables	Participants with a Self-Reported Diagnosis of Depression n = 3217 (%)		Participants without a Self-Reported Diagnosis of Depression n = 39,063 (%)		*p*-Value
Gender					<0.001
Female	2209 (68.67%)		19,130 (48.97%)		
Male	1008 (31.33%)		19,933 (51.03%)		
Age group					<0.001
16–24 years old	52 (1.61%)		3726 (9.54%)		
25–44 years old	846 (26.30%)		17,535 (44.89%)		
45–64 years old	2319 (72.09%)		17,802 (45.57%)		
Marital status					<0.001
Single	779 (24.21%)		13,041 (33.38%)		
Married	1627 (50.58%)		22,207 (56.85%)		
Widowed	265 (8.24%)		799 (2.05%)		
Separated/Divorced	546 (16.97%)		3016 (7.72%)		
Level of education					<0.001
Without studies	272 (8.46%)		1361 (3.48%)		
Primary	636 (19.77%)		4023 (10.30%)		
Secondary or PT	1948 (60.55%)		24,672 (63.16%)		
University	361 (11.22%)		9007 (23.06%)		
Nationality					<0.001
Spanish	3068 (95.37%)		35,789 (91.62%)		
Foreign	149 (4.63%)		3274 (8.38%)		
Size of town of residence					0.71
<10,000 inhabitants	693 (21.54%)		8527 (21.83%)		
≥10,000 inhabitants	2524 (78.46%)		30,536 (78.17%)		
Social class					<0.001
Classes I and II	384 (11.94%)		8531 (21.84%)		
Classes III and IV	967 (30.06%)		13,561 (34.72%)		
Classes V and VI	1866 (58.00%)		16,971 (43.44%)		
Body Mass Index					<0.001
Underweight	64 (1.99%)		917 (2.35%)		
Normal weight	1221 (37.95%)		18,922 (48.44%)		
Overweight	1189 (36.96%)		13,448 (34.43%)		
Obese	743 (23.10%)		5776 (14.78%)		
Current smoking habit					<0.001
Yes	1198 (37.24%)		12,146 (31.09%)		
No	2019 (62.76%)		26,917 (68.91%)		
Consumption of alcoholic beverages in the past 12 months prior to the survey					<0.001
Yes	1638 (50.92%)		26,911 (68.89%)		
No	1579 (49.08%)		12,152 (31.11%)		
Physical activity in main activity					<0.001
Physically active	443 (13.77%)		7647 (19.58%)		
Not physically active	2774 (86.23%)		31,416 (80.42%)		
Number of days in the last 7 days when the respondent had walked for at least 10 min at a time (maximum 7 days)	M	SD	M	SD	<0.001
	4.29	2.92	4.64	2.81	

PT = Professional Training; M = mean; SD = Standard Deviation.

**Table 2:** Frequency of food consumption and diet quality according to depressive status in participants aged from 16 to 64 years (N = 42,280) (2011–2017).

Variables	Participants with a Self-Reported Diagnosis of Depression n = 3217 (%)	Participants without a Self-Reported Diagnosis of Depression n = 39,063 (%)	*p*-Value
Frequency of consumption of bread or grains			<0.001
Never or hardly ever	108 (3.36)	718 (1.84)	
Less than once a week	73 (2.27)	671 (1.72)	
Once or twice a week	129 (4.00)	1564 (4.00)	
Three or more times a week, but not daily	254 (7.90)	3579 (9.16)	
Daily	2653 (82.47)	32,531 (83.28)	
Frequency of consumption of leafy greens, salads and vegetables			<0.001
Never or hardly ever	61 (1.90)	469 (1.20)	
Less than once a week	99 (3.07)	925 (2.37)	
Once or twice a week	403 (12.53)	4548 (11.64)	
Three or more times a week, but not daily	1228 (38.17)	16,061 (41.12)	
Daily	1426 (44.33)	438 (43.67)	
Frequency of fresh fruit (excluding juices) consumption			<0.001
Never or hardly ever	156 (4.85)	1315 (3.37)	
Less than once a week	166 (5.16)	1491 (3.82)	
Once or twice a week	305 (9.48)	4017 (10.28)	
Three or more times a week, but not daily	597 (18.56)	8519 (21.81)	
Daily	1993 (61.95)	23,721 (60.72)	
Frequency of consumption of dairy products (milk, cheese, yoghurt)			<0.001
Never or hardly ever	124 (3.85)	990 (2.54)	
Less than once a week	76 (2.36)	803 (2.06)	
Once or twice a week	125 (3.89)	1525 (3.90)	
Three or more times a week, but not daily	240 (7.46)	3497 (8.95)	
Daily	2652 (82.44)	32,248 (82.55)	
Frequency of meat (chicken, beef, pork, lamb, etc.) consumption			<0.001
Never or hardly ever	56 (1.74)	443 (1.13)	
Less than once a week	130 (4.04)	693 (1.77)	
Once or twice a week	972 (30.22)	9585 (24.54)	
Three or more times a week, but not daily	1807 (56.17)	24,380 (62.41)	
Daily	252 (7.83)	3962 (10.15)	
Frequency of legumes consumption			0.01
Never or hardly ever	91 (2.83)	956 (2.45)	
Less than once a week	370 (11.50)	4236 (10.85)	
Once or twice a week	1890 (58.75)	23,970 (61.36)	
Three or more times a week, but not daily	820 (25.49)	9507 (24.34)	
Daily	46 (1.43)	394 (1.00)	
Frequency of consumption of cold meats and cuts			<0.001
Never or hardly ever	449 (13.96)	3868 (9.90)	
Less than once a week	647 (20.11)	6305 (16.14)	
Once or twice a week	951 (29.56)	11,627 (29.76)	
Three or more times a week, but not daily	784 (24.37)	11,694 (29.94)	
Daily	386 (12.00)	5569 (14.26)	
Frequency of consumption of sweets (biscuits, pastries, jams, cereals with sugar, sweets, etc.)			<0.001
Never or hardly ever	574 (17.84)	5528 (14.15)	
Less than once a week	596 (18.53)	6550 (16.77)	
Once or twice a week	626 (19.46)	8453 (21.64)	
Three or more times a week, but not daily	515 (16.01)	7596 (19.44)	
Daily	906 (28.16)	10,936 (28.00)	
Frequency of consumption of soft drinks with sugar			<0.001
Never or hardly ever	1606 (49.92)	15,503 (39.69)	
Less than once a week	643 (19.99)	7911 (20.25)	
Once or twice a week	383 (11.91)	6920 (17.72)	
Three or more times a week, but not daily	261 (8.11)	4247 (10.87)	
Daily	324 (10.07)	4482 (11.47)	
Diet quality			<0.001
Poor diet quality	98 (3.05)	1060 (2.71)	
Diet in need of improvement	2114 (65.71)	27,448 (70.27)	
Good diet quality	1005 (31.24)	10,555 (27.02)	

**Table 3:** Frequency of food consumption by people with a self-reported diagnosis of depression in Spain aged from 16 to 64 years by year of survey (N = 3217) (2011–2017).

Variables	2011/2012 n = 930 (%)	2014 n = 1168 (%)	2017 n = 1119 (%)	*Β*	R^2^	*p*-Value
Frequency of consumption of bread or grains						
Never or hardly ever	35 (3.76)	40 (3.43)	33 (2.95)	−0.14	0.99	0.07
Less than once a week	24 (2.58)	30 (2.57)	19 (1.70)	−0.15	0.76	0.33
Once or twice a week	47 (5.06)	38 (3.25)	44 (3.93)	−0.19	0.38	0.58
Three or more times a week, but not daily	57 (6.13)	109 (9.33)	88 (7.86)	0.29	0.29	0.64
Daily	767 (82.47)	951 (81.42)	935 (83.56)	0.18	0.26	0.66
Frequency of consumption of leafy greens, salads and vegetables						
Never or hardly ever	22 (2.36)	24 (2.05)	15 (1.34)	−0.17	0.95	0.14
Less than once a week	36 (3.87)	31 (2.65)	32 (2.86)	−0.17	0.60	0.44
Once or twice a week	125 (13.44)	149 (12.76)	129 (11.53)	−0.32	0.97	0.10
Three or more times a week, but not daily	309 (33.23)	452 (38.70)	467 (41.73)	1.42	0.97	0.10
Daily	438 (47.10)	512 (43.84)	476 (42.54)	−0.76	0.94	0.15
Frequency of fresh fruit (excluding juices) consumption						
Never or hardly ever	67 (7.21)	41 (3.51)	48 (4.29)	−0.49	0.56	0.46
Less than once a week	45 (4.84)	49 (4.19)	72 (6.43)	0.27	0.48	0.52
Once or twice a week	88 (9.46)	103 (8.82)	114 (10.19)	0.12	0.28	0.64
Three or more times a week, but not daily	136 (14.62)	242 (20.72)	219 (19.57)	0.83	0.58	0.45
Daily	594 (63.87)	733 (62.76)	666 (59.52)	−0.72	0.93	0.18
Frequency of consumption of dairy products (milk, cheese, yoghurt)						
Never or hardly ever	43 (4.62)	43 (3.68)	38 (3.40)	−0.20	0.91	0.19
Less than once a week	17 (1.83)	32 (2.74)	27 (2.41)	0.10	0.40	0.57
Once or twice a week	30 (3.23)	50 (4.28)	45 (4.02)	0.13	0.52	0.49
Three or more times a week, but not daily	47 (5.05)	105 (8.99)	88 (7.86)	0.47	0.48	0.51
Daily	793 (85.27)	938 (80.31)	921 (82.31)	−0.49	0.35	0.60
Frequency of meat (chicken, beef, pork, lamb, etc.) consumption						
Never or hardly ever	22 (2.37)	13 (1.11)	21 (1.88)	−0.08	0.15	0.75
Less than once a week	40 (4.30)	50 (4.28)	40 (3.57)	−0.12	0.77	0.32
Once or twice a week	304 (32.69)	346 (29.62)	322 (28.78)	−0.65	0.90	0.20
Three or more times a week, but not daily	493 (53.01)	671 (57.45)	643 (57.46)	0.74	0.75	0.33
Daily	71 (7.63)	88 (7.54)	93 (8.31)	0.11	0.65	0.40
Frequency of consumption of legumes						
Never or hardly ever	39 (4.19)	33 (2.83)	19 (1.70)	−0.42	1.00	0.03
Less than once a week	113 (12.15)	131 (11.21)	126 (11.26)	−0.15	0.71	0.36
Once or twice a week	538 (57.85)	662 (56.68)	690 (61.66)	0.63	0.54	0.48
Three or more times a week, but not daily	222 (23.87)	330 (28.25)	268 (23.95)	0.01	0.00	0.99
Daily	18 (1.94)	12 (1.03)	16 (1.43)	−0.09	0.31	0.62
Frequency of consumption of cold meats and cuts						
Never or hardly ever	195 (20.96)	132 (11.30)	122 (10.90)	−1.68	0.78	0.31
Less than once a week	198 (21.29)	250 (21.41)	199 (17.79)	−0.58	0.72	0.35
Once or twice a week	255 (27.42)	367 (31.42)	329 (29.40)	0.33	0.25	0.67
Three or more times a week, but not daily	151 (16.24)	289 (24.74)	344 (30.74)	2.42	0.99	0.06
Daily	131 (14.09)	130 (11.13)	125 (11.17)	−0.49	0.74	0.34
Frequency of consumption of sweets (biscuits, pastries, jams, cereals with sugar, sweets, etc.)						
Never or hardly ever	245 (26.34)	180 (15.41)	149 (13.31)	−2.17	0.87	0.24
Less than once a week	158 (16.99)	212 (18.15)	226 (20.20)	0.54	0.98	0.10
Once or twice a week	131 (14.09)	238 (20.38)	257 (22.97)	1.48	0.95	0.15
Three or more times a week, but not daily	99 (10.64)	217 (18.58)	199 (17.78)	1.19	0.67	0.39
Daily	297 (31.94)	321 (27.48)	288 (25.74)	−1.03	0.94	0.16
Frequency of consumption of soft drinks with sugar						
Never or hardly ever	527 (56.67)	539 (46.15)	540 (48.26)	−1.40	0.57	0.45
Less than once a week	139 (14.95)	256 (21.92)	248 (22.16)	1.20	0.77	0.31
Once or twice a week	94 (10.11)	140 (11.99)	149 (13.31)	0.53	0.99	0.06
Three or more times a week, but not daily	63 (6.77)	115 (9.84)	83 (7.42)	0.11	0.04	0.87
Daily	107 (11.50)	118 (10.10)	99 (8.85)	−0.44	1.00	0.02
Diet quality						
Poor diet quality	25 (2.69)	45 (3.85)	28 (2.50)	−0.03	0.02	0.92
Diet in need of improvement	529 (56.88)	798 (68.32)	787 (70.33)	2.24	0.86	0.24
Good diet quality	376 (40.43)	325 (27.83)	304 (27.17)	−2.21	0.79	0.31

*p*-value is for trend.

**Table 4:** Association between diet quality and sociodemographic, lifestyle, and health-related variables in people with a self-reported diagnosis of depression in Spain aged from 16 to 64 years (N = 3217) (2011–2017).

Variables	Individuals with a Self-Reported Diagnosis of Depression (N = 3217)					
	Poor/Need Improvement Diet (n = 2212)					
	n (%)		OR (CI 95%)	*p*-Value	ORa (CI 95%) ^1^	*p*-Value
Gender						
Female	1444 (65.37%)		Reference		Reference	
Male	768 (76.19%)		1.70 (1.43–2.01)	<0.001	1.47 (1.23–1.76)	<0.01
Age group (years)						<0.01 <0.001
16–24	45 (86.54%)		3.71 (1.67–8.27)	<0.01	3.05 (1.24–6.95)	
25–44	697 (82.39%)		2.70 (2.22–3.29)	<0.001	2.32 (1.89–2.86)	
45–64	1470 (63.39%)		Reference		Reference	
Marital status						0.34 0.27 <0.01
Single	621 (79.72%)		0.72 (0.56–0.93)	0.01	0.88 (0.67–1.15)	
Married	1061 (65.21%)		Reference		Reference	
Widowed	152 (57.36%)		1.20 (0.97–1.48)	0.09	1.13 (0.91–1.41)	
Separated/divorced	378 (69.23%)		2.10 (1.71–2.57)	<0.001	1.41 (1.13–1.75)	
Social class						
Social classes I and II	265 (69.01%)		0.99 (0.78–1.26)	0.94		
Social classes III and IV	656 (67.84%)		0.93 (0.80–1.11)	0.46		
Social classes V and VI	1291 (69.19%)		Reference			
Level of education						
Without studies	188 (69.12%)		0.94 (0.71–1.23)	0.63		
Primary	407 (63.99%)		0.74 (0.62–0.90)	<0.01		
Secondary or PT	1374 (70.53%)		Reference			
University	243 (67.31%)		0.86 (0.68–1.10)	0.22		
Nationality						
Spanish	2094 (68.25%)		Reference	<0.01		
Foreigner	118 (79.19%)		1.78 (1.18–2.65)			
Size of town of residence						
≤10,000 inhabitants	462 (66.67%)		0.89 (0.74–1.06)	0.18		
>10,000 inhabitants	1750 (69.33%)		Reference			
Body mass index						
Underweight	52 (81.25%)		1.75 (0.92–3.32)	0.09		
Normal weight	870 (71.25%)		Reference			
Overweight	797 (67.03%)		0.80 (0.65–0.97)	0.02		
Obesity	493 (66.35%)		0.82 (0.69–0.98)	0.03		
Self-perceived health status						
Very good	90 (74.38%)		1.28 (0.83–1.95)	0.26		
Good	599 (67.68%)		0.92 (0.77–1.11)	0.37		
Fair	920 (69.49%)		Reference			
Poor	541 (69.38%)		0.99 (0.81–1.22)	0.96		
Very poor	152 (64.14%)		0.79 (0.59–1.06)	0.10		
Current smoking habit						<0.001
Yes	931 (77.71%)		2.01 (1.71–2.37)	<0.001	1.70 (1.43–2.02)	
No	1281 (63.45%)		Reference		Reference	
Consumption of alcoholic beverages in the past 12 months prior to the survey						<0.01
Yes	1184 (72.28%)		Reference		Reference	
No	1028 (65.10%)		0.72 (0.32–0.83)	<0.001	0.80 (0.69–0.94)	
Physical activity in main activity						
Physically active in main activity	299 (67.49%)		0.94 (0.75–1.16)	0.54		
Not physically active in main activity	1913 (68.96%)		Reference			
Physical activity during leisure time						
Yes	1130 (66.90%)		Reference			
No	1082 (70.81%)		1.20 (1.03–1.40)	0.02		
Number of days in the last 7 days when the respondent walked for at least 10 min at a time	M	SD	0.96 (0.93–0.98)	<0.01	0.95 (0.92–0.97)	<0.001
	4.17	2.95				

PT = Professional Training; M = mean; SD = Standard Deviation; OR = Odds Ratio; ORa = Odds Ratio Adjusted for all sociodemographic, lifestyle and health-related variables; CI95% = Confidence Interval; n = number of people with a poor or improvable diet quality; ^1^ the variables included in the final multivariate-adjusted model were: gender, age group, marital status, current smoking habit, consumption of alcoholic beverages in the past 12 months prior to the survey and number of days in the last 7 days when the respondent had walked for at least 10 min at a time; Hosmer–Lemeshow test χ^2^ = 3.31, *p* = 091; Nagelkerke’s R^2^: 0.10; *p*-value < 0.001.

## Data Availability

The data presented in this study are available as Supplementary Material (File S1: Research data).

## Supplementary Materials

The following are available online at https://www.mdpi.com/2072-6643/13/1/106/s1, Table S1: Criteria to define the score for each item of the Spanish Health Eating Index (SHEI), File S1: Research Data.
