# Supplementary material for: Diet Quality and Sociodemographic, Lifestyle, and Health-Related Determinants among People with Depression in Spain: New Evidence from a Cross-Sectional Population-Based Study (2011–2017)
Source: Nutrients. 2020 Dec 30;13(1):106. doi: 10.3390/nu13010106 (PMC7823268; doi:10.3390/nu13010106)
Supplement: Supplementary file 1 [file nutrients-13-00106-s001.zip › nutrients-1047255-supplementary/Supplementary Table S1.docx]

**Supplementary Table S1.** Criteria to define the score for each item of the Spanish Health Eating Index (SHEI).

| **Criteria to define the score for each item of the Spanish Health Eating Index (SHEI)** | | | | | |
| --- | --- | --- | --- | --- | --- |
| **Variables** | Criteria for a maximum score of 10 | Criteria for a maximum score of 7.5 | Criteria for a maximum score of 5 | Criteria for a maximum score of 2.5 | Criteria for a maximum score of 0 |
| Daily |  |  |  |  |  |
| Bread or grains | Daily | Three or more times a week, but not daily | Once or twice a week | Less than once a week | Never or almost never |
| Leafy greens, salads and vegetables | Daily | Three or more times a week, but not daily | Once or twice a week | Less than once a week | Never or almost never |
| Fresh fruit (excluding juices) | Daily | Three or more times a week, but not daily | Once or twice a week | Less than once a week | Never or almost never |
| Dairy products (milk, cheese, yoghurt) | Daily | Three or more times a week, but not daily | Once or twice a week | Less than once a week | Never or almost never |
| Weekly consumption |  |  |  |  |  |
| Meat (chicken, beef, pork, lamb, etc.) | Once or twice a week | Three or more times a week, but not daily | Less than once a week | Daily | Never or almost never |
| Legumes | Once or twice a week | Three or more times a week, but not daily | Less than once a week | Daily | Never or almost never |
| Occasional consumption |  |  |  |  |  |
| Cold meats and cuts | Never or almost never | Less than once a week | Once or twice a week | Three or more times a week, but not daily | Daily |
| Sweets (biscuits, pastries, jams, cereals with sugar, sweets, etc.) | Never or almost never | Less than once a week | Once or twice a week | Three or more times a week, but not daily | Daily |
| Soft drinks with sugar | Never or almost never | Less than once a week | Once or twice a week | Three or more times a week, but not daily | Daily |
| Variety | 2 points if participant achieve each of the daily recommendations, 1 point if participant achieve each of the weekly recommendations. | | | | |

Each item scored from 0 to 10 depending to the criteria of the Spanish Health Eating Index (SHEI) [1], which 10 points means that the recommendations proposed by the Spanish Society of Community Nutrition (SSCN) [2].

Supplementary references

1. Norte Navarro, A.; Ortiz Moncada, R. Spanish diet quality according to the healthy eating index. *Nutr*. *Hosp*. **2011**, *26*, 330–336, doi: 10.1590/S0212-16112011000200014.
2. Spanish Society of Community Nutrition (SSCN). Healthy dietary guidelines (2014). Available online: <http://www.nutricioncomunitaria.org/es/otras-publicaciones> (accessed on 21 December 2020).
